# Sulfamic acid pyromellitic diamide-functionalized MCM-41 as a multifunctional hybrid catalyst for melting-assisted solvent-free synthesis of bioactive 3,4-dihydropyrimidin-2-(1*H*)-ones

**DOI:** 10.1038/s41598-021-89572-y

**Published:** 2021-05-27

**Authors:** Ehsan Valiey, Mohammad G. Dekamin, Zahra Alirezvani

**Affiliations:** Pharmaceutical and Heterocyclic Compounds Research Laboratory, Department of Chemistry, Iran University of Science and Technology, 16846-13114 Tehran, Iran

**Keywords:** Catalysis, Environmental chemistry, Green chemistry, Materials chemistry, Medicinal chemistry, Organic chemistry, Supramolecular chemistry, Chemical synthesis

## Abstract

This study introduces a practical approach to fabricate a novel hybrid acidic catalyst, namely sulfamic acid pyromellitic diamide-functionalized MCM-41 (MCM-41-APS-PMDA-NHSO_3_H). Various techniques such as FTIR, TGA, XRD, BET, FESEM, and EDX were used to confirm its structural characteristics. The efficiency of the new MCM-41-APS-PMDA-NHSO_3_H organosilica nanomaterials, as a heterogenous nanocatalyst, was examined in the synthesis of biologically active 3,4-dihydropyrimidin-2-(1*H*)-one derivatives under solvent-free conditions. It was found that the nanoporous MCM-41-APS-PMDA-NHSO_3_H, demonstrating acidic nature and high surface area, can activate all the Biginelli reaction components to afford desired 3,4-dihydropyrimidin-2-(1*H*)-ones under solvent-free conditions in short reaction time. Furthermore, easy and quick isolation of the new introduced hybrid organosilica from the reaction mixture as well as its reusability with negligible loss of activity in at least five consecutive runs are another advantages of this green protocol.

## Introduction

In recent decades, the synthesis and use of mesoporous structures have received much attention. The M41S family consists mainly of silica, SiO_2_. Silica has certain advantages such as high chemical and thermal stability, large number of silanol (Si − OH) groups and simplicity of operation, which have made it an appropriate and well-known support in the chemical industry. MCM-41 became the most attractive member of the M41S family due to its ordered structure and special properties such as exceptional high surface area (> 1000 m^2^ g^−1^) and narrow pore-size distribution (1.5–10 nm)^1–4^. These properties have made MCM-41 as an appropriate nanomaterial support for metal oxides^5^, heteropoly acids^6^, metal–ligand complexes^7,8^, etc. to immobilize catalytic active centers^9–13^ as well as to develop more efficient drug delivery systems^14–18^, sensors^19^, degradation inhibitors in polymer industry^20^, adsorbents of organic pollutants^21–23^. However, the acidic strength of the pure MCM-41 is relatively weak, which hinders its catalysis applications. Therefore, modification of its surface can lead to the formation of solid acids with high uniformity, which are regularly prepared by covalent anchoring of various organic moieties with proper functional groups in a mesoporous material or replacing of Si atoms by other tetra-, tri- and di-valent metals such as Al, B, Fe, Mn, Zn, etc^16,24–40^. Hence, covalent anchoring of both sulfamic and pyromellitic acids in the pore walls of MCM-41 can significantly enhance the catalytic capabilities of the designed catalyst.

On the other hand, solvent-free organic synthesis (SFOS) has been emerged as an effective tool for the rapid preparation of various organic compounds especially biologically active molecules during recent years^41^. In fact, solvent-free conditions obviously form a liquid phase on heating of the reaction mixture with solid substrates. This melting mentions the eutectic mixture with temperature fusion below the melting points of the reactants. These solvent-free protocols have many advantages including the products are sufficiently pure which does not require further purification or recrystallization; the reactions are sometimes rapid as compared to conditions using often toxic solvents; functional group protection–deprotection can be avoided, and sometimes the use of solvent-free conditions is more inexpensive^42,43^. Furthermore, the use of multicomponent reactions (MCRs) allows formation of densely functionalized organic molecules such as dihydropyrimidinones (DHPMs) in a simple synthetic procedure^43–47^. Hence, the simultaneous use of solid acids, solvent-free conditions and MCRs would be very beneficial to prepare high-value organic compounds as well as address green chemistry principles^48^.

**Scheme 1:**
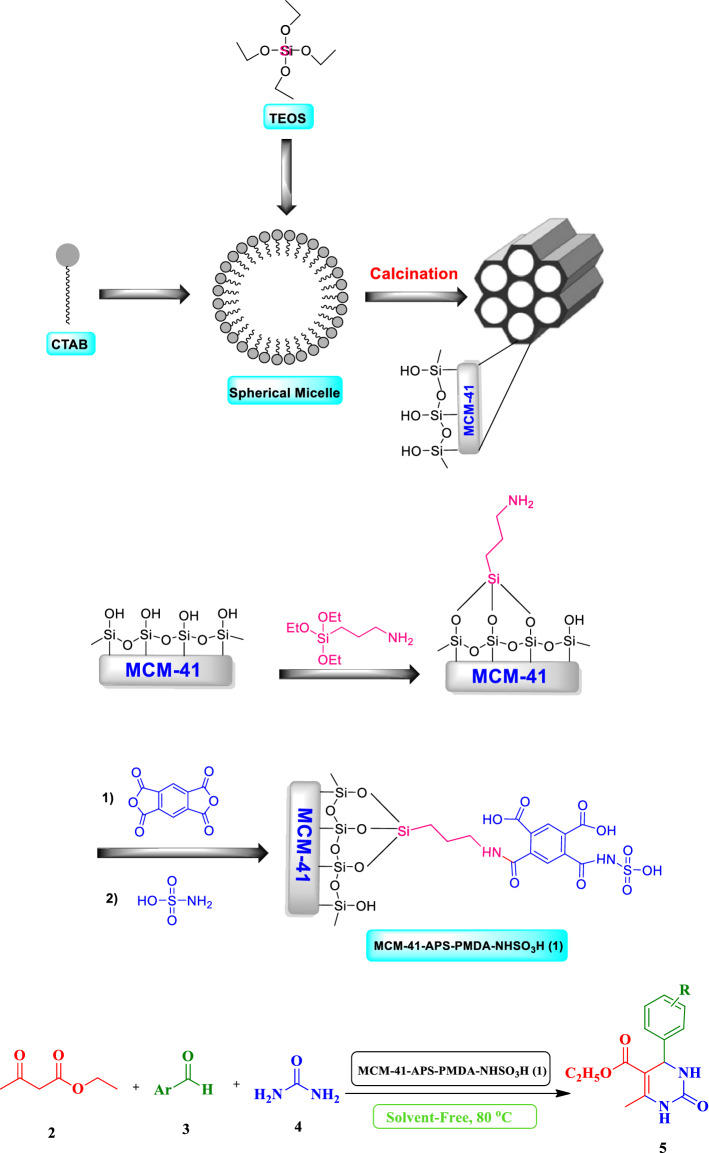
Schematic preparation of MCM-41-APS-PMDA-NHSO_3_H (**1**) for the three-component condensation of ethyl acetoacetate (**2**), aldehydes (**3**), urea (**4**) to afford 3,4-dihydropyrimidin-2-(1*H*)-one derivatives (**5**).

In the past few decades, dihydropyrimidinones (DHPMs) and their derivatives, as an important class of heterocyclic compounds, have stimulated interest in medicinal chemistry due to their diverse biological activities^48–51^. Theses pyrimidine-containing heterocycles are present significantly in natural products or synthetic organic compounds such as natural marine polycyclic guanidine alkaloids, the kinesin Eg5 inhibitor Monastrol, BACE-1 inhibitor to prevent Alzheimer’s disease, bioprobes and fluorescent sensors^51–55^. Due to important properties of DHMPs, different methods using Brønsted or Lewis acids catalysts have been developed for the synthesis of DHPMs by the Biginelli reaction in recent years^55–59^. Among these catalytic systems, the immobilization of the catalytic active centers on a wide range of solid polymer supports, especially silica, can improve the efficiency of the relevant method^59–63^. In continuation of our ongoing efforts towards developing of more efficient heterogeneous catalysts for different MCRs^63–70^, we wish herein to introduce preparation and characterization of the new hybrid sulfamic acid pyromellitic diamide-functionalized MCM-41 (MCM-41-APS-PMDA-NHSO_3_H) nanomaterials. Also, its catalytic activity was investigated in the three-component synthesis of 3,4-dihydropyrimidin-2-(1*H*)-one derivatives from aromatic aldehydes, ethyl acetoacetate and urea (Scheme 1). To the best of our knowledge, there is not any report for the use of sulfamic acid pyromellitic diamide grafted on the surface of MCM-41, as a heterogeneous nanocatalyst, for the synthesis of Biginelli 3,4-dihydropyrimidin-2-(1*H*)-one derivatives.

## Results and discussion

### Characterization of the MCM-41-APS-PMDA-NHSO_3_H nanomaterials (1)

**Figure 1:**
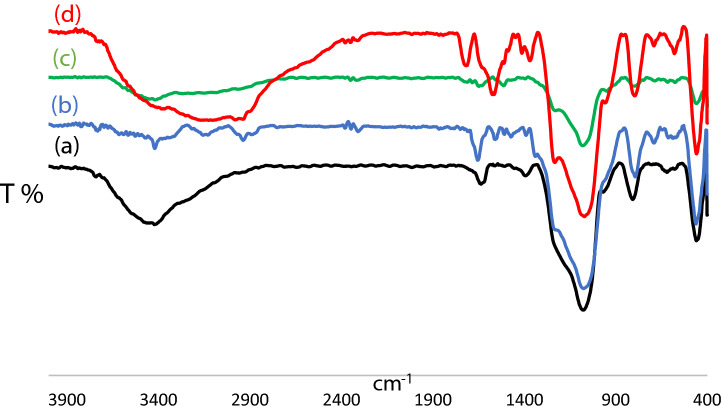
FTIR spectra of the MCM-41 (**a**), MCM-41-APS (**b**), MCM-41-APS-PMDA (**c**) and MCM-41-APS-PMDA-NHSO_3_H (**d**) (**1**).

The as prepared MCM-41-APS-PMDA-NHSO_3_H nanomaterial was analyzed using different spectroscopic, microscopic and analytical methods as well as porsiometric and porometric techniques including FTIR, EDX, XRD, FESEM, TGA and BET experiments. The FTIR spectra of MCM-41 (a), MCM-41-APS (b) MCM-41-APS-PMDA (c) and MCM-41-APS-PMDA-NHSO_3_H are show in Fig. 1. The nano-ordered MCM-41 shows a band in the 3443 cm^−1^ region that is due to the presence of both Si–OH and OH groups of the adsorbed water molecules on its surface (Fig. 1a)^71^. Furthermore, the band corresponded to Si–O–Si bonds for MCM-41 and all subsequent modifications are observed around 1228–1062 cm^−1^. Also, the signals in the regions of 1600 cm^−1^ and 2883 cm^−1^ are attributed to the symmetric vibrations of NH_2_ and the asymmetric vibrations of C–H of 3-APTS, respectively (Fig. 1b). On the other hand, the decrease in signal intensity of the OH groups of MCM-41 surface confirms that the MCM-41 substrate has been modified by the covalent bonding of the 3-APTS linker. In addition, the observed broad band at 3604–2923, 1716 and 1569 cm^−1^ are attributed to the stretching vibrations of the pyromellitic acid and its amide derivative (Fig. 1c). Also, the characteristic band observed at 1365 and 1066 cm^−1^ are assigned to the asymmetric and symmetric S=O stretching vibration of the SO_3_H group (Fig. 1d).

**Figure 2:**
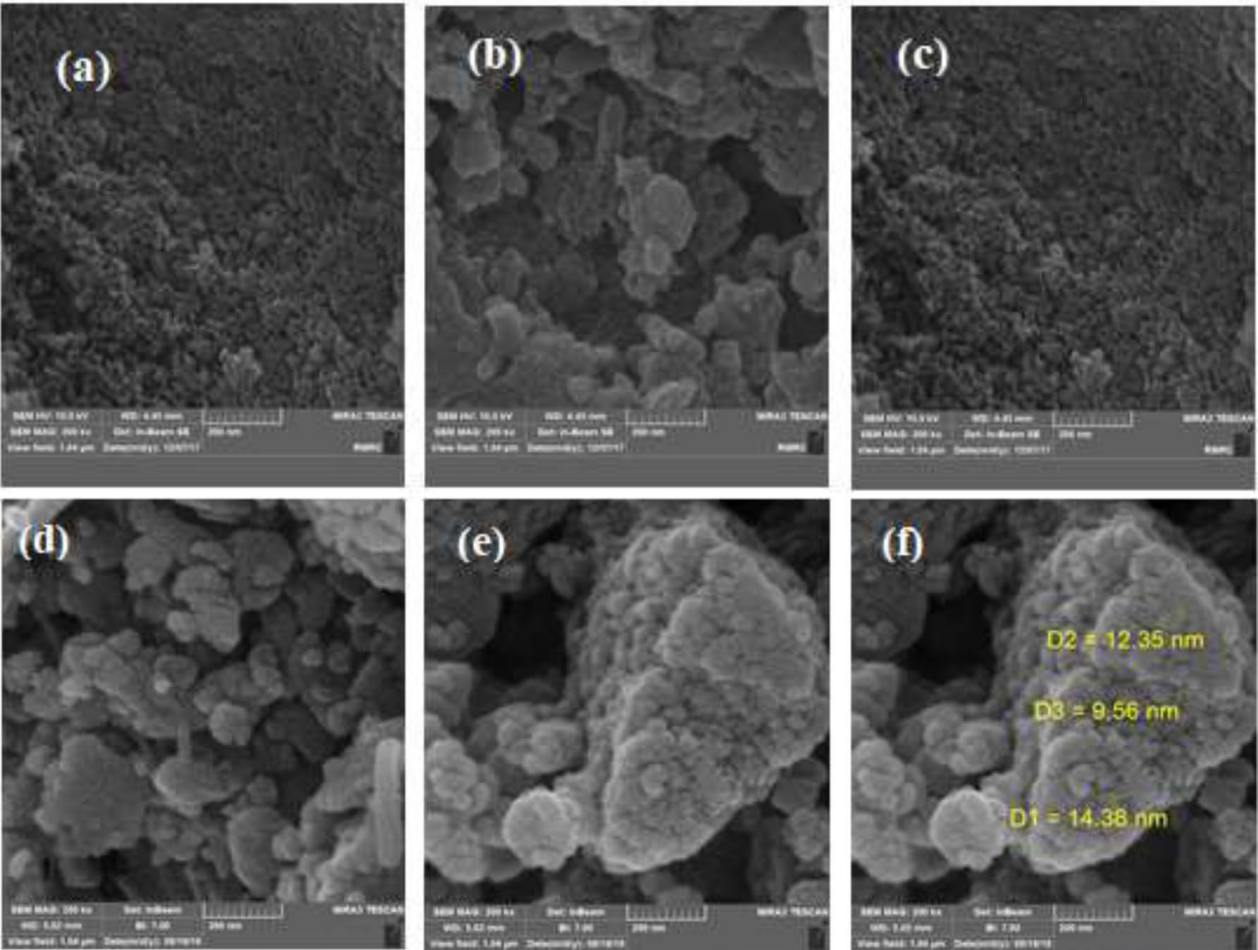
FESEM images of the MCM-41 (**a**–**c**) and the MCM-41-APS-PMDA-NHSO_3_H (**1**, **d**–**f**) materials.

Also, the morphology and textural properties of the MCM-41and MCM-41-APS-PMDA-NHSO_3_H (**1**) were examined using field emission scanning electron microscopy (FESEM). As shown in Fig. 2, the morphological distinction between the pure MCM-41 (2a–c images) and MCM-41-APS-PMDA-NHSO_3_H (**1**, 2d–f images) demonstrate grafting of the *N*-carbonyl sulfamic acid pyromellitic diamide moiety on the outer surface of MCM-41 support.

**Figure 3:**
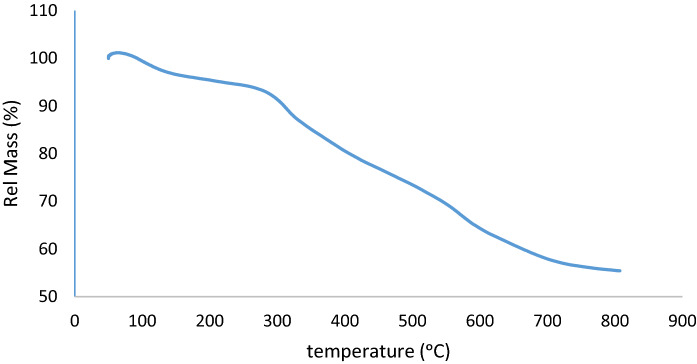
TGA analysis of the MCM-41-APS-PMDA-NHSO_3_H materials (**1**).

On the other hand, the thermogravimetric analysis (TGA) of the MCM-41-APS-PMDA-NHSO_3_H (**1**) are shown in Fig. 3. The TGA curve of MCM-41-APS-PMDA-NHSO_3_H shows three distinct steps of weight loss. In the first step, 10% weight loss between 50 °C and 150 °C can be attributed to the absorbed water or solvent molecules held in the pores of the MCM-41-APS-PMDA-NHSO_3_H nanomaterial. The second weight loss between 150 and 350 °C is due to decomposition of the grafted organic *N*-carbonyl sulfamic acid pyromellitic diamide moiety. Also, the third weight loss (17%) between 380 and 600 °C can be related to the conversion of silanol (Si–OH) groups to siloxane (Si–O–Si) bridges. These results also indicate that the *N*-carbonyl sulfamic acid pyromellitic diamide moiety has successfully been grafted onto the surface of MCM-41.

**Figure 4:**
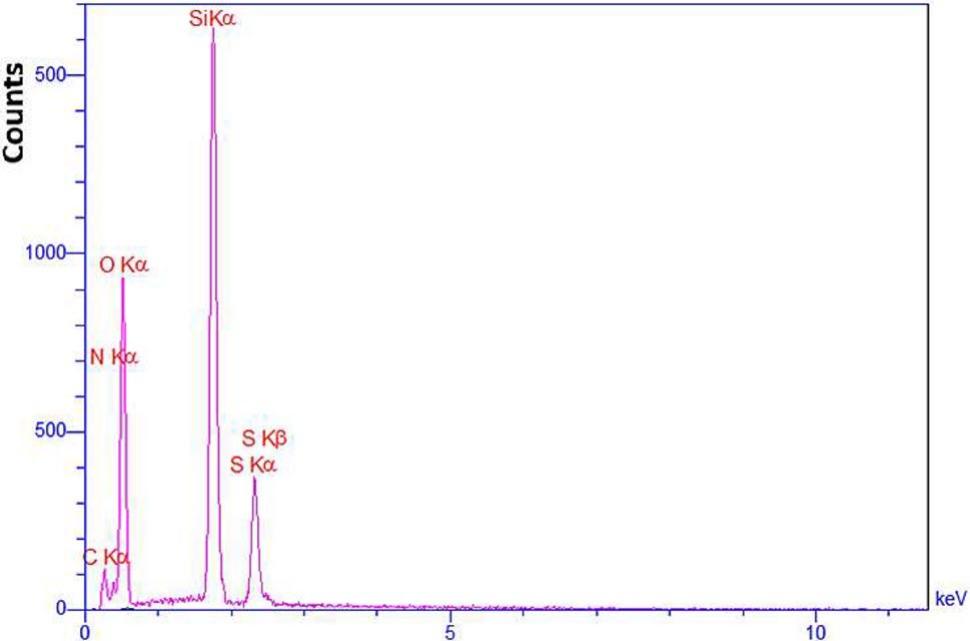
EDX spectra of the MCM-41-APS-PMDA-NHSO_3_H materials (**1**).

As shown in Fig. 4, the energy-dispersive X-ray (EDX) spectra of the MCM-41-APS-PMDA-NHSO_3_H (**1**) verified the presence of Si (11.61%), C (14.89%), O (57.63%), N (12.90%), and S (2.98%), respectively.

**Figure 5:**
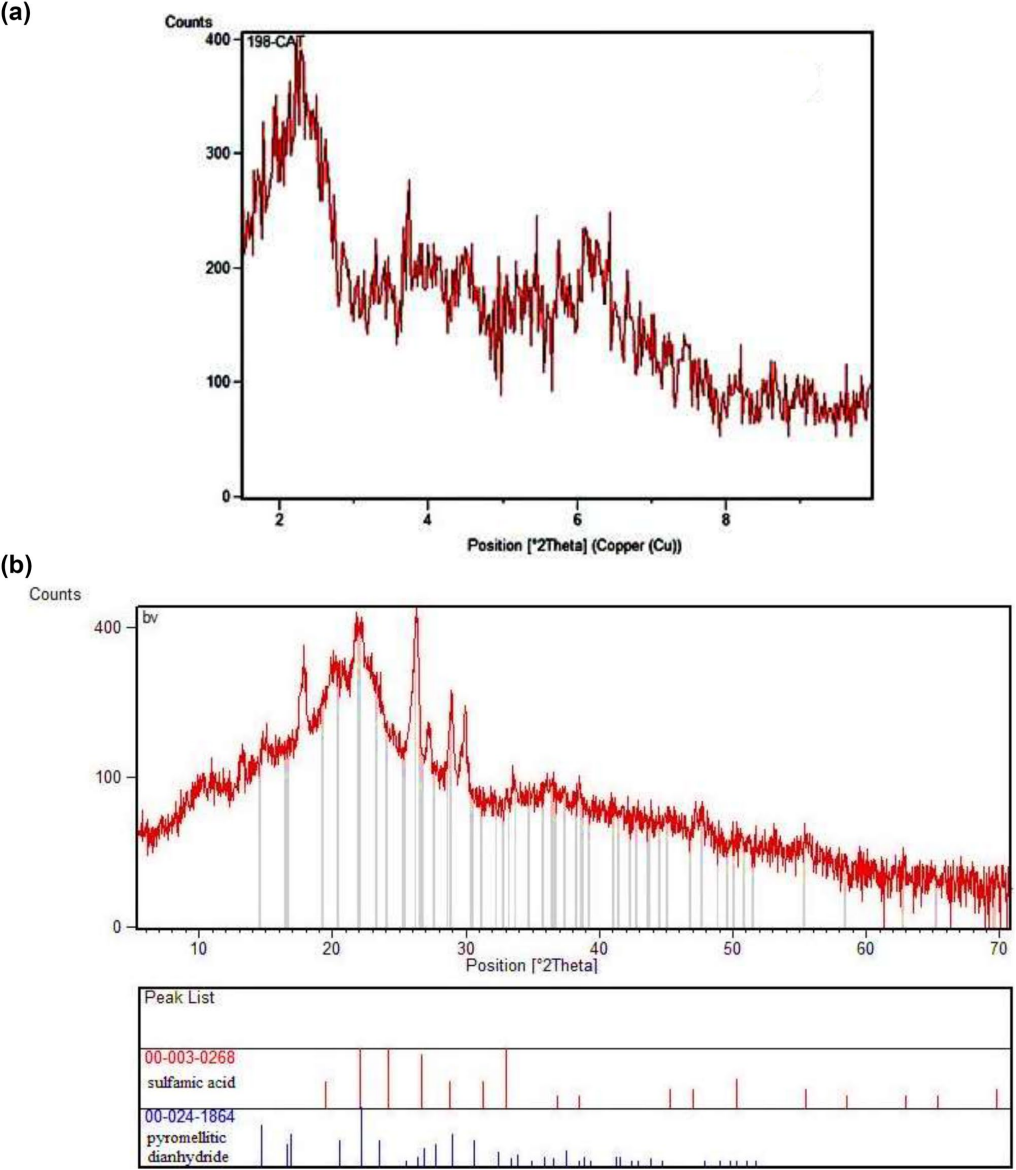
Low angle (**a**) and wide angle (**b**) XRD patterns of the hybrid MCM-41-APS-PMDA-NHSO_3_H nanocatalyst (**1**).

Furthermore, the powder XRD pattern of the MCM-41-APS-PMDA-NHSO_3_H (**1**, Fig. 5a) shows low angle reflections of (d100), (d110) and (d200) at 2θ = 2.77°, 4.67° and 5.13°, respectively. These plates confirm the formation of a hexagonal mesoporous structure with regular particle size and pores, which indicates its structure is similar to the mesoporous MCM-41 precursor. On the other hand, the observed peaks in the wide angle XRD pattern are in well agreement with both Joint Committee on Powder Diffraction Standards (JCPDS) card no 00-003-0268 (sulfamic acid) and 00-024-1864 (pyromellitic dianhydride). These data also demonstrate successful grafting of the the organic moieties onto the surface of nanocatalyst **1**. Indeed, the diffraction signals observed at 2θ = 14.0°, 19.0°, 23.0°, 25.0°, 26.0°, 29.3° illustrates the formation of MCM-41-APS-PMDA-NHSO_3_H (Fig. 5b).

**Figure 6:**
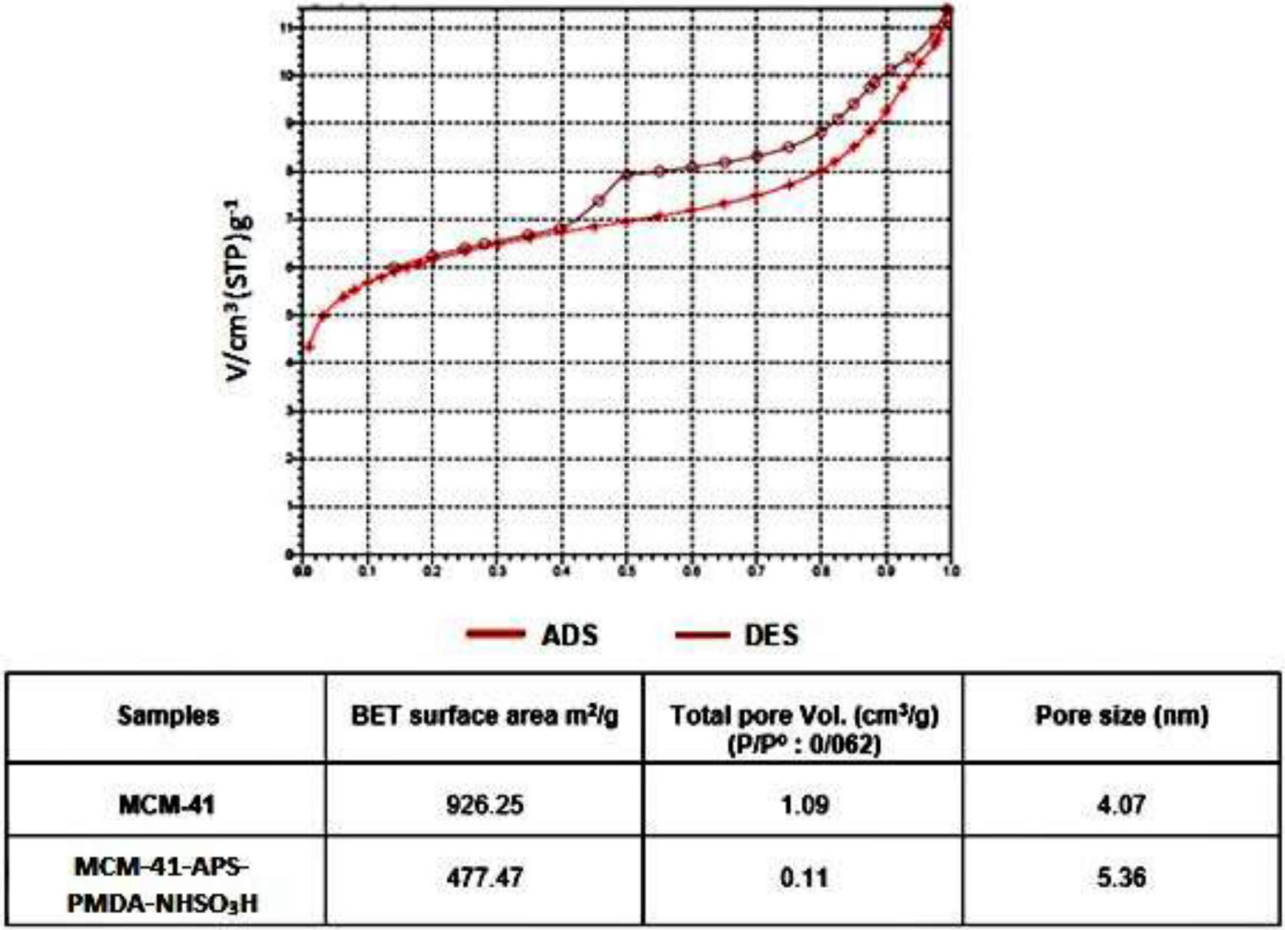
Adsorption/desorption isotherm of the MCM-41-APS-PMDA-NHSO_3_H nanocatalyst (**1**).

Figure 6 demonstrates the N_2_ adsorption/desorption isotherms of the MCM-41, MCM-41-APS-PMDA-NHSO_3_H. Isotherm type V was recognizable for MCM-41-APS-PMDA-NHSO_3_H with hysteresis loop. The table shows the parameters such as pore volume as well as average pore diameter in MCM-41 and the nanocatalyst **1**. In fact, grafting of APS-PMDA-NHSO_3_H groups through the (3-aminopropyl) triethoxysilane and pyromellitic acid linkers reduces both surface area and pore volume whereas increases pore diameter of the nanocatalyst **1**.

### Investigation of the catalytic activity of the MCM-41-APS-PMDA-NHSO_3_H nanocatalyst (1) for the synthesis of 3,4-dihydropyrimidinones 5a–k

To evaluate the catalytic activity of the MCM-41-APS-PMDA-NHSO_3_H nanomaterials (**1**) in the synthesis of 3,4-dihydropyrimidin-2(1*H*)-ones, the reaction of ethyl acetoacetate (**2**, 1 mmol), 4-chlorobenzaldehyde (**3a**, 1 mmol) and urea (**4**, 1.2 mmol) was investigated as the model reaction under different conditions. A systematic study was performed to optimize different parameters affecting of the model reaction such as solvent, catalyst loading and temperature. The results are summarized in Table 1. The results of using different polar and non-polar solvents as well as solvent-free conditions showed that the model reaction proceeded very well with lower catalyst **1** loading under solvent-free conditions at 80 °C in shorter reaction time (Table 1, entries 1–11). These findings encouraged us to perform the model reaction under solvent-free conditions in further optimization reactions (entries 12–14). Indeed, by further reducing of the catalyst loading, lower yields of the desired product ethyl 4-(4-chlorophenyl)-6-methyl-2-oxo-1,2,3,4-tetrahydropyrimidine-5-carboxylate (**5a**) were obtained under similar conditions even over longer times. On the other hand, it is noteworthy that a very low yield of the desired product **5a** was obtained in the absence of the MCM-41-APS-PMDA-NHSO_3_H nanomaterials (**1**). Therefore, these results strongly confirmed the role of MCM-41-APS-PMDA-NHSO_3_H (**1**) to promote the synthesis of 3,4-dihydropyrimidin-2(1*H*)-ones under solvent-free conditions. Hence, 15 mg of catalyst **1** under solvent-free conditions at 80 °C were selected as the optimal conditions for the next experiments.

**Table 1:** Optimization of conditions in the model reaction of ethyl acetoacetate (**2**), 4-chlorobenzaldehyde (**3a**), urea (**4**) under different conditions in the presence of MCM-41-APS-PMDA-NHSO_3_H (**1**).^a^

Entry	Catalyst loading (mg)	Solvent	Temperature (°C)	Time (min)	Yield^b^ (%) **5a**
1	20	MeOH	r.t	180	26
2	20	EtOH	Reflux	90	69
3	20	CH_2_Cl_2_	Reflux	90	45
4	20	CH_3_CN	60	120	78
5	20	DMF	Reflux	100	62
6	20	Toluene	Reflux	150	48
7	20	Et_2_O	r.t	240	35
8	20	CHCl_3_	60	120	75
9	20	EtOH/H_2_O (1:2)	Reflux	55	77
10	20	EtOH/H_2_O (1:1)	Reflux	70	73
11	15	Solvent-free	80	35	95
12	10	Solvent-free	80	65	69
13	5	Solvent-free	80	90	60
14	2	Solvent-free	80	120	57
15	0	Solvent-free	80	180	15

^a^Reaction conditions: ethyl acetoacetate (**2**, 1 mmol), 4-chlorobenzaldehyde (**3a**, 1 mmol), urea (**4**, 1.2 mmol), MCM-41-APS-PMDA-NHSO_3_H (**1**) and solvent (2 ml, if not otherwise stated).

The optimized conditions were developed to different carbocyclic or heterocyclic aromatic aldehydes affording other 3,4-dihydropyrimidin-2(1*H*)-one derivatives. The results are summarized in Table 2. Noticeably, the desired products **5a–k** were obtained in high to excellent yields. In fact, aldehydes **3** bearing electron-withdrawing groups on their aromatic ring generally react faster compared to those having electron-donating groups. These results clearly confirm the appropriate catalytic activity of the MCM-41-APS-PMDA-NHSO_3_H hybrid nanomaterials (**1**) to promote the Biginelli condensation of a wide range of aldehydes with ethyl acetoacetate and urea.

**Table 2:** Scope of the Biginelli condensation for the synthesis of 3,4-dihydropyrimidin-2-(1*H*)-ones catalyzed by MCM-41-APS-PMDA-NHSO_3_H (1)^a^.

Entry	Aldehyde 3	Product 5	Time (min)	Yield (%)^b^	mp °C (Obs.)	mp °C (Lit.)
1	4-ClC_6_H_4_–		35	95	210–211	210–211^72^
2	C_6_H_5_–		55	87	235–236	234–236^73^
3	4-NO_2_C_6_H_4_–		50	80	204	224–227^74^
4	3-NO_2_C_6_H_4_–		60	82	293–295	204^75^
5	4-CH_3_OC_6_H_4_–		55	89	201–203	202–204^76^
6	2-ClC_6_H_4_–		60	90	211–213	211–213^75^
7	4-OHC_6_H_4_–		55	82	234–236	233–235^77^
8	2-C_4_H_3_S–		45	84	212–214	210–212^78^
9	4-Me_2_NC_6_H_4_–		45	92	213–215	213–215^79^
10	4-FC_6_H_4_–		60	85	180	180^80^
11	4-OH-3-MeO-C_6_H_3_–		40	84	188–190	188.5^81^

^a^Reaction conditions: ethyl acetoacetate (2, 1 mmol), aldehydes (3a–k, 1 mmol), urea (4, 1.2 mmol), MCM-41-APS-PMDA-NHSO_3_H (1, 15 mg) under solvent-free conditions at 80 °C.

^b^Isolated yields were reported.

**Scheme 2:**
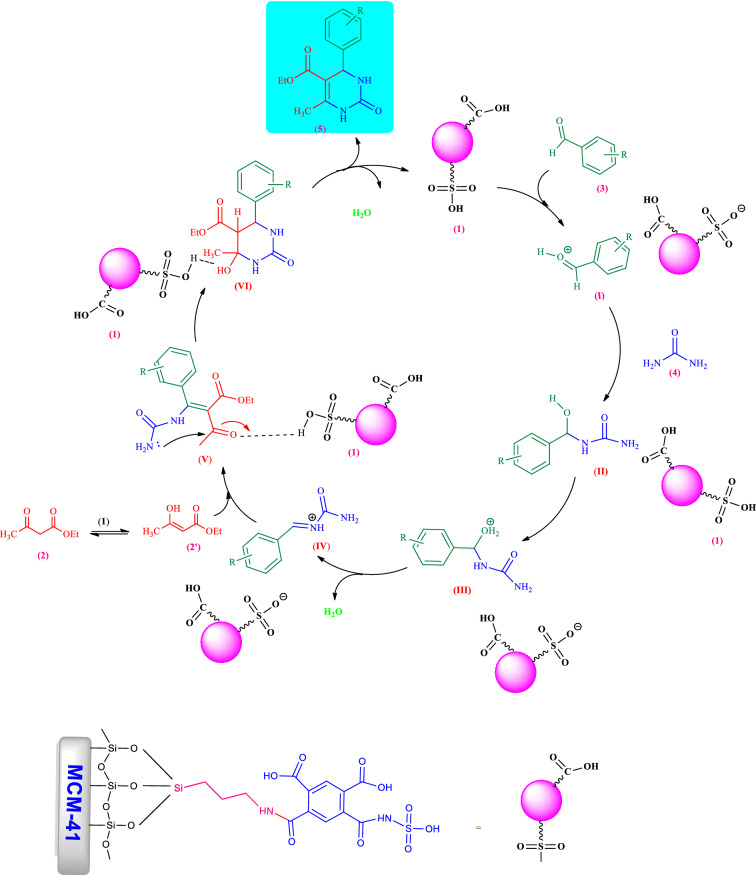
Proposed mechanism for the synthesis of 3,4-dihydropyrimidin-2(1*H*)-ones catalyzed by MCM-41 (MCM-41-APS-PMDA-NHSO_3_H (**1**).

According to the above results and observations, the following mechanism can be proposed for the synthesis of 3,4-dihydropyrimidin-2(1*H*)-ones derivatives catalyzed by the MCM-41-APS-PMDA-NHSO_3_H nanocatalyst (**1**, Scheme 2). Firstly, MCM-41-APS-PMDA-NHSO_3_H (**1**) activates the carbonyl group of aromatic aldehyde **3** for the addition of urea **4** on it to form intermediate (**II**). Followed by dehydration of this intermediate, the corresponding iminium intermediate (**IV**) is formed. Then, intermediate (**V**) is produced after addition of the enol form of ethyl acetoacetate (**2′**) on the intermediate (**IV**). Subsequent cyclization of the intermediate (**V**) and final dehydration of intermediate (**VI**) afford corresponding 3,4-dihydropyrimidin-2(1*H*)-ones **5**. Furthermore, eliminated water molecules during the catalytic cycle can be adsorbed on the surface of catalyst **1** and facilitate the reaction.

**Figure 7:**
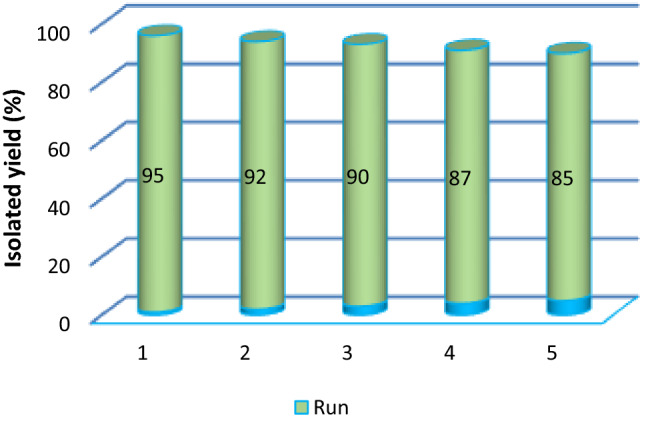
Reusability of the heterogeneous acidic nanocatalyst MCM-41-APS-PMDA-NHSO_3_H (**1**) for the synthesis of **5a**.

As a part of our study, the heterogeneous solid acid catalyst **1** was separated from the model reaction mixture after its completion, washed several times with EtOH, and then dried in an oven at 60 °C for 1.5 h. The recycled catalyst **1** was reused in four consecutive model reaction under optimized conditions. The results are shown in Fig. 7. Interestingly, only a very little decrease in the catalytic activity of the MCM-41 (MCM-41-APS-PMDA-NHSO_3_H (**1**, approx. 10%) was observed.

To illustrate the merits of catalytic activity of the new MCM-41-APS-PMDA-NHSO_3_H organosilica nanomaterials, as a heterogeneous solid acid, its efficiency has been compared with some of the previously reported catalysts for the preparation of **5a** (Table 3). The results illustrate that this study is actually superior to other cases in terms of desired product yield, amount of catalyst loading, reaction time, working under solvent-free conditions, avoiding of the use of corrosive or expensive reagents and transition metals, and the reusability of the catalyst for at least five consecutive runs.

**Table 3:** Comparison of the catalytic activity of the MCM-41-APS-PMDA-NHSO_3_H (**1**) with other catalysts.

Entry	Catalyst	Amount of catalyst loading	Solvent	Temp. (°C)	Time (min)	Yield (%)	References
1	PPF-SO_3_H	250 mg	EtOH	Reflux	480	81	^73^
2	PANI-FeCl_3_	200 mg	CH_3_CN	Reflux	1440	83	^82^
3	Fe_3_O_4_/PAA-SO_3_H	60 mg	Solvent-free	RT	120	90	^83^
4	H_2_SO_4_.Silica gel	30 mol ⁒(47 mg)	Solvent-free	60 °C	120	89	^84^
5	Zr(H_2_PO_4_)_2_	7 mol ⁒(20 mg)	Solvent-free	90 °C	60	92	^85^
6	**MCM-41-APS-PMDA-NHSO**_**3**_**H**	**15 mg**	**Solvent-**f**ree**	**80 °C**	**35**	**96**	**This work**

## Experimental section

### General information

All chemicals were purchased from Merck or Aldrich chemical companies. Melting points were measured using an Electrothermal 9100 device and are unmodified. Characterization of the new hybrid nanocatalyst **1** was performed by FESEM TESCAN-MIRA3, EDX Numerix DXP-X10P, Shimadzu FTIR-8400S and TGA Bahr Company STA 504. The XRD pattern of the catalyst was obtained using a TW 1800 diffractometer with Cu Ka radiation (λ = 1.54050 Å). The analytical thin layer chromatography (TLC) experiments was performed using Merck 0.2 mm silica gel 60F-254Al-plates. All compounds are known and well characterized by FTIR and ^1^H NMR (500 MHz, Bruker DRX-500 Avance, in DMSO-d_6_ at ambient temperature) spectroscopy.

### General procedure for preparation of the MCM-41

Nano-ordered mesoporous silica MCM-41 was prepared by the hydrothermal synthesis and according to known reported method^86^. 2.70 g of diethyl amine was dissolved in 42 mL deionized water at room temperature. The mixture was stirred for 10 min, then 1.47 g of cetyltrimethylammonium bromide (CTAB) was added and the obtained mixture was stirred for 30 min until a clear solution was produced. Next, 2.10 g tetraethyl orthosilicate (TEOS) was gently added and by dropwise addition of HCl solution (1 M), the pH of the mixture was fixed at 8.5 to afford the final precipitate. The resulting mixture was stirred for 2 h and then the resulting white precipitate was filtered and washed with 100 mL of distilled water. Afterward, the obtained white solid was dried at 45 °C for 12 h and finally the sample was calcined at 550 °C with the rate of 2 °C/min for 5 h.

### ***General procedure for preparation of the MCM-41-APS-PMDA-NHSO***_***3***_***H (1)***

In a 200 mL round button flask, (3-aminopropyl) triethoxysilane (3-APTS, 0.15 mmol, d = 0.946 g/mL) was added to a mixture of MCM-41 (0.15 g) in dry toluene (15 mL) under stirring and reflux conditions. After 8 h, the obtained white MCM-41-APS solid was filtered, and washed with toluene and CHCl_3_ several times to remove any excess of the 3-APTS linker. The MCM-41-APS-NH_2_ solid was heated in an oven at 80 °C for 8 h. Next, dried MCM-41-APS-NH_2_ solid (0.15 g) and pyromellitic dianhydride (0.15 g) were dispersed in dry THF (30 mL) and the obtained mixture was stirred at r.t for 1 h. Following this, triethylamine (TEA, 0.10 g) was added to the obtained mixture. Then the mixture was stirred at r.t for 24 h under N_2_ atmosphere. Afterward, the obtained solid was filtered off and washed with toluene and EtOH (2 mL), respectively, for several times. The as-prepared solid having an anhydride functional group was first dispersed in dry toluene (20 mL) and then triethylamine (0.10 g) and sulfamic acid (0.10 g) were added. The obtained mixture was stirred under N_2_ atmosphere and reflux conditions for 36 h. Finally, the white solid was filtered, washed with EtOH (2 mL) for several times and dried in oven at 60 °C for 8 h. The preparation schematic route of the MCM-41-APS-PMDA-NHSO_3_H nanomaterials **(1)** has been shown in Scheme 1.

### ***General procedure for the synthesis of 3,4-dihydropyrimidinone-2-(1H)-ones*** 5a–k ***catalyzed by the MCM-41-APS-PMDA-NHSO***_***3***_***H (1)***

In a 5 mL round-bottom flask, a mixture of ethyl acetoacetate (**2**, 1 mmol), aldehydes (**3**, 1 mmol), urea (**4**, 1.2 mmol) and MCM-41-APS-PMDA-NHSO_3_H (**1**, 15 mg) was heated at 80 °C under solvent-free conditions for times indicated in Table 2. The progress of the reactions was monitored by TLC (Eluent: EtOAc: n-hexane, 1:3). After completion of the reaction, 96% EtOH (3 mL) was added to the mixture. The heterogeneous catalyst was then separated by filtration and the filtrate was allowed to cool over time to give pure crystals of the desired 3,4-dihydropyrimidinones **5a–k**. The separated catalyst was suspended in EtOH (2 mL) and stirred at r.t for 30 min. Then, it was filtered off and dried in an oven at 60 °C for 1.5 h to be used for next runs.

## Conclusions

In summary, we have developed an efficient and practical synthetic methodology for the preparation of 3,4-dihydropyrimidin-2(1*H*)-ones using sulfamic acid pyromellitic diamide-functionalized MCM-41 (MCM-41-APS-PMDA-NHSO_3_H), as a heterogeneous multifunctional hybrid catalyst, under solvent-free conditions. Low catalyst loading, high to quantitative yield of the desired products and compatibility with various functional groups as well as easy and quick isolation of the products from the reaction mixture and reusability of the novel solid acidic hybrid organosilica with negligible loss of its activity are the main advantages of this procedure. Further works to expand and apply MCM-41-APS-PMDA-NHSO_3_H nanomaterials in different organic transformations is ongoing in our laboratory and would be presented in due courses.
